# Novel *VPS13B* Mutations in Three Large Pakistani Cohen Syndrome Families Suggests a Baloch Variant with Autistic-Like Features

**DOI:** 10.1186/s12881-015-0183-0

**Published:** 2015-06-25

**Authors:** Muhammad Arshad Rafiq, Claire S Leblond, Muhammad Arif Nadeem Saqib, Akshita K. Vincent, Amirthagowri Ambalavanan, Falak Sher Khan, Muhammad Ayaz, Naseema Shaheen, Dan Spiegelman, Ghazanfar Ali, Muhammad Amin-ud-din, Sandra Laurent, Huda Mahmood, Mehtab Christian, Nadir Ali, Alanna Fennell, Zohair Nanjiani, Gerald Egger, Chantal Caron, Ahmed Waqas, Muhammad Ayub, Saima Rasheed, Baudouin Forgeot d’Arc, Amelie Johnson, Joyce So, Muhammad Qasim Brohi, Laurent Mottron, Muhammad Ansar, John B Vincent, Lan Xiong

**Affiliations:** Molecular Neuropsychiatry & Development Laboratory, Campbell Family Mental Health Research Institute, Centre for Addiction and Mental Health, 250 College Street, Toronto, ON M5T 1R8 Canada; CHUM Research Center - Notre Dame Hospital, Montreal, Canada; Department of Biochemistry, Quaid-I-Azam University, and Pakistan Medical Research Council, Islamabad, Pakistan; The Lahore Institute for Research and Development, Lahore, Punjab Pakistan; University of Education, Township Campus, College Road, Lahore, Punjab Pakistan; Department of Biotechnology, University of Azad Jammu and Kashmir, P.O. Box 13100, Muzaffarabad, Pakistan; Dept: zoology, University of Education, Lahore, Campus Dera Ghazi Khan, Punjab Pakistan; Ma Ayesha Memorial Centre, Karachi, Pakistan; Institute of Human Genetics, Medical University of Graz, Graz, A-8010 Austria; Hôpital Rivière-des-Prairies, Montreal, Canada; Département de Psychiatrie, Université de Montréal, Montreal, Canada; Division of Developmental Disabilities, Department of Psychiatry, Queen’s University, Kingston, ON Canada; Autism Institute, Karachi, Pakistan; Research Centre, Montreal Mental Health University Institute, 7331, rue Hochelaga, Montréal, QC H1N 3 V2 Canada; The Fred A. Litwin Family Centre in Genetic Medicine, University Health Network and Mount Sinai Hospital, Toronto, Canada; The Centre for Addiction and Mental Health, Toronto, Canada; Department of Laboratory Medicine and Pathobiology, University of Toronto, Toronto, Canada; Sir Cowasji Jehangir Institute of Psychiatry, Hyderabad, Sindh Pakistan; Department of Psychiatry, University of Toronto, Toronto, ON Canada; Currently at: Department of Physiology and Experimental Medicine (PEM), Hospital for Sick Children, Toronto, ON Canada; Currently at: Montreal Neurological Institute, McGill University, Montreal, QC Canada

## Abstract

**Background:**

Cohen Syndrome (COH1) is a rare autosomal recessive disorder, principally identified by ocular, neural and muscular deficits. We identified three large consanguineous Pakistani families with intellectual disability and in some cases with autistic traits.

**Methods:**

Clinical assessments were performed in order to allow comparison of clinical features with other *VPS13B* mutations. Homozygosity mapping followed by whole exome sequencing and Sanger sequencing strategies were used to identify disease-related mutations.

**Results:**

We identified two novel homozygous deletion mutations in *VPS13B*, firstly a 1 bp deletion, NM_017890.4:c.6879delT; p.Phe2293Leufs*24, and secondly a deletion of exons 37-40, which co-segregate with affected status. In addition to COH1-related traits, autistic features were reported in a number of family members, contrasting with the “friendly” demeanour often associated with COH1. The c.6879delT mutation is present in two families from different regions of the country, but both from the Baloch sub-ethnic group, and with a shared haplotype, indicating a founder effect among the Baloch population.

**Conclusion:**

We suspect that the c.6879delT mutation may be a common cause of COH1 and similar phenotypes among the Baloch population. Additionally, most of the individuals with the c.6879delT mutation in these two families also present with autistic like traits, and suggests that this variant may lead to a distinct autistic-like COH1 subgroup.

**Electronic supplementary material:**

The online version of this article (doi:10.1186/s12881-015-0183-0) contains supplementary material, which is available to authorized users.

## Background

Intellectual disability (ID) affects ~1 % of the population [1], however for up to 80 % of ID patients the underlying etiology is unknown. Chromosomal aberrations are thought to be the most common cause [2]. In a cohort of 1170 unselected patients referred for developmental delay or ID, Cohen syndrome (COH1) [MIM# 216550] was found among several common genetic diagnoses for ID; and with a frequency of 0.7 %, COH1 is ranked right after Fragile-X syndrome (1.2 %) among patients with clear diagnosis of ID [2]. COH1 is an autosomal recessively inherited ID disorder, first described by Cohen and colleagues in 1973 [3]. Kivitie-Kallio and Norio [4] proposed that the essential features for a COH1 diagnosis include non-progressive psychomotor retardation, motor clumsiness, microcephaly, distinctive facial features, childhood hypotonia, joint laxity, chorioretinal dystrophy, myopia and granulocytopenia [4]. After much debate on these diagnostic criteria in ethnically diverse groups, newly amended diagnostic guidelines were given in which a child with significant learning difficulties must show at least two of the three features: typical facial gestalt, pigmentary retinopathy or neutropenia [5, 6].

To date, COH1 has been mainly attributed to mutations in the *VPS13B* gene (MIM# 607817) among patients from many different ethnic backgrounds [7–15].

For approximately 70 % of COH1 patients both mutated alleles of *VPS13B* were detected, whereas in approximately 20-30 % of the cases a single heterozygous mutation was found [8, 10]. A total of 159 *VPS13B* gene mutations (HGMD, 2013.4) have been identified which have since led to further modification of the clinical diagnostic criteria for differentiation between Cohen and Cohen-like syndrome [8, 14, 16–18]. In the present study, we describe the ascertainment and clinical assessment of three large consanguineous families from Pakistan segregating ID in an autosomal recessive pattern. We also describe the mapping by autozygosity of the disease locus for each family to 8q22, followed by screening and mutation detection in *VPS13B* through either candidate gene sequencing or whole exome sequencing.

## Methods

### Patients and families

**Fig. 1:**
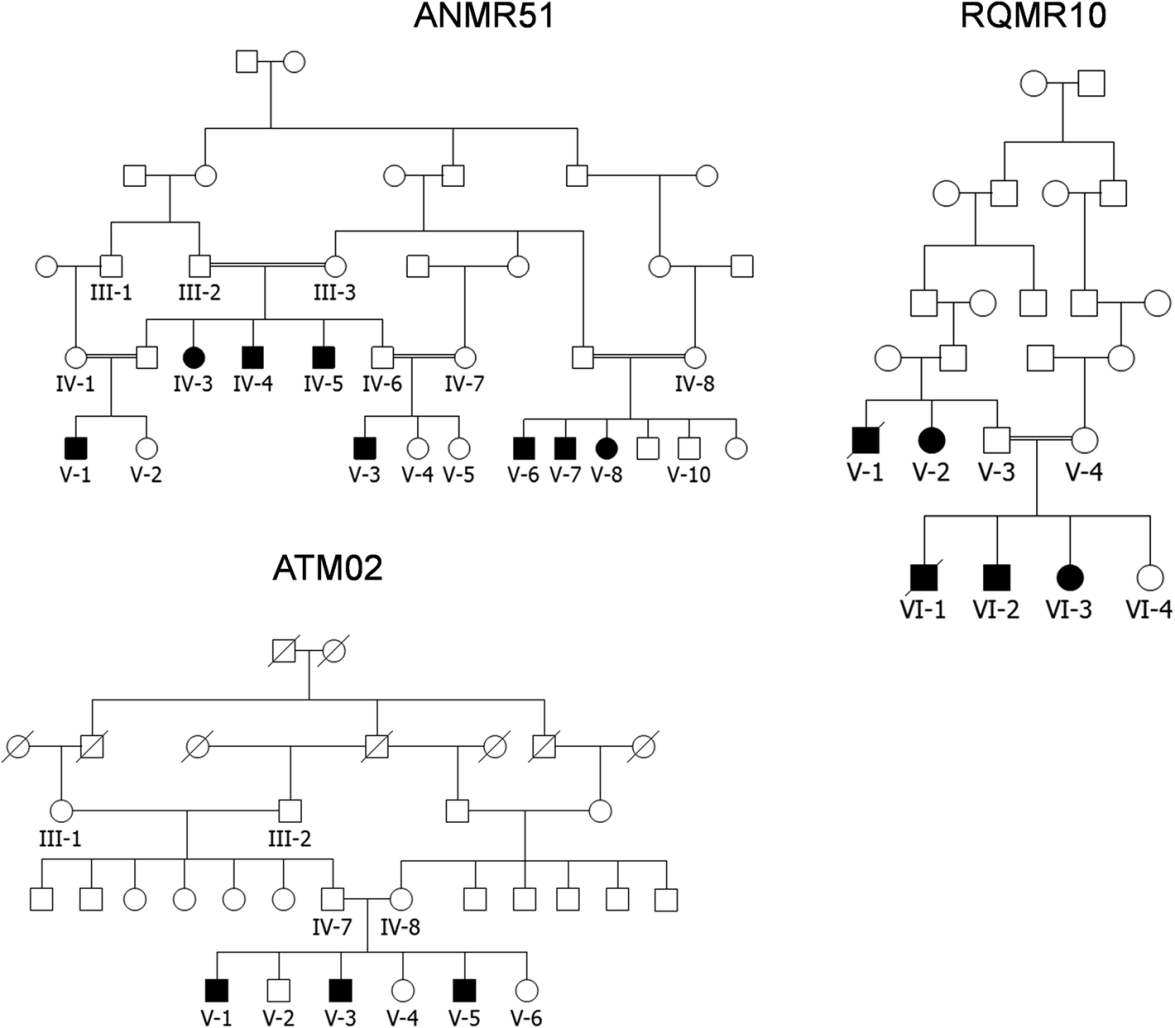
Pedigrees of the three Pakistani families. For family RQMR10, although the degree of relatedness between the parents of V-1, V-2, and V-3 could not be established, marriages within this rural community are strictly within the clan system, and thus they are almost certainly related. ATM02 is part of a much larger pedigree segregating ID, with 18 affected individuals, however only members of the portion indicated here was assessed in this study. This full pedigree is shown in Additional files 1, 2, 3 and 4

Two of the Pakistani families (RQMR10 and ANMR51) were initially identified through referral to the study on genetics of autosomal recessive ID through collaborations between the Lahore Institute of Research & Development (LIRD), Pakistan, and the Centre for Addiction & Mental Health (CAMH), Toronto, Canada, and between Quaid-i-Azam University, Islamabad and CAMH. These two families are from Dera Ghazi Khan District, Punjab Province, and from Naushahro Feroze district in Sindh Province, respectively. The third Pakistani family (ATM02) was identified and recruited as a recessive form of pervasive developmental disorder (PDD) from Dadu District, Sindh Province, through collaboration between the Sir Cowasji Jehangir Institute of Psychiatry (SCJIP), Hyderabad, Pakistan and CHUM Research Center (CRCHUM), University of Montreal, Canada. Research Ethics Board approval was obtained from the involved institutions (LIRD, Quaid-i-Azam University, CAMH, SCJIP and CRCHUM), and written informed consent was obtained from the parents/guardians and other family members for publication of this article and any accompanying images, in compliance with the Helsinki Declaration. The pedigrees (Fig. 1) were constructed by interviewing different family members. 293 healthy Pakistani controls were also recruited, after written informed consent was given, so that variants could be checked for frequency in an ethnically matched population.

### Clinical investigation of families RQMR10 and ANMR51

All affected family members were examined clinically, with special attention to neurological, psychological, ophthalmological, skeletal, behavioral and dental anomalies. One affected member from RQMR10 family (VI-2) was subjected to fundoscopic examination, in order to investigate the ophthalmological condition associated with the disease in that family. Complete biochemical testing of blood of affected as well as several unaffected individuals was performed for both families, in particular to confirm or exclude neutropenia — one of the characteristic features of COH1.

### Clinical investigation of ATM02 family

The three affected brothers were examined by local clinicians, including a psychiatrist, a clinical psychologist and a neurologist, and evaluated retrospectively using the Childhood Autism Rating Scale (CARS) [19]. One of the affected individuals was also available for additional clinical tests: including routine blood/urine test, biochemistry test (serum TSH, B12 and folic acid, CPK, and random blood glucose test) and brain MRI. Their clinical synopsis, CARS ratings, lab test results and diagnosis have been further reviewed and validated by three pediatric psychiatrists (C.C., B.F.A., L.M.) from Montreal.

### DNA extraction, genotyping, homozygosity mapping and sequencing

Genomic DNA was extracted from either whole blood or from saliva of affected and unaffected family members, by following standard protocols. For RQMR10, DNA samples from three affected (V-2, VI-2, VI-3) and two unaffected individuals (V-3, V-4), were genotyped using the Affymetrix Genome-Wide Human SNP 6.0 microarray at The Centre for Applied Genomics (TCAG), Toronto. DNA samples from eight affected individuals (IV-3, IV-4, IV-5, V-1, V-3, V-6, V-7, V-8) and one unaffected individual (V-10) from ANMR51 were genotyped using the AffymetrixGeneChip Mapping 500 K NspI array, at the London Regional Genomics Centre (LRGC, University of Western Ontario). For ATM02, the three affected individuals (V-1, V-3, V-5) and four unaffected individuals (IV-7, IV-8, V-4 and V-6) were genotyped using the Illumina HumanOmniExpress BeadChip at the Genome Quebec and McGill University Innovation Centre.

For RQMR10 and ANMR51 homozygosity-by-descent (HBD) or autozygosity mapping and copy number variation (CNV) analysis was performed using dChip software [20, 21]. For ATM02, KaryoStudio program from Illumina Inc. was used to exclude cytogenetic abnormalities, and HBD mapping was performed by using the HomozygosityMapper [22].

For RQMR10 and ANMR51, direct Sanger sequencing of *VPS13B* was performed, based on homozygosity mapping results, for all 62 exons and splice junctions of *VPS13B*. PCR primers were designed using Primer3 (version 0.4.0). PCR amplicons were sequenced at TCAG. Sequencing data were aligned to the annotated human genome sequence (hg19) using the BLAT tool of the UCSC Genome Browser, and variants confirmed by checking the sequencing chromatograms. Once identified, the inheritance pattern of the variant was checked across the pedigree for cosegregation with the phenotype. Potential candidate variants were further validated in Pakistani population controls through Sanger sequencing.

For ATM02 family, whole exome capturing and sequencing in two affected individuals (V-1 and V-5) were performed using Agilent SureSelect™ 50 Mb Human All Exon Kit and sequenced by HiSeq2000 at the Genome Quebec and McGill University Innovation Centre. Sequence data was processed and aligned using a bioinformatic pipeline including FASTQC, BWA [23], GATK [24] and ANNOVAR programs [25]. An in-house script was created to generate familial variant segregation files. Potential candidate variants were further validated in additional family members, and Pakistani population controls through Sanger sequencing.

### Deletion break-point mapping

A PCR-based approach was used in order to map the deletion breakpoints in family ANMR51. Sets of primers were designed using Primer 3 (Primer3 software, version 0.4.0) for regions flanking the putative deleted exons (exons 37–40). PCR amplification was performed using a forward primer from the closest proximal amplicon and a reverse primer from the closest distal set that that amplified in affected individuals, in order to identify the junction fragment. The junction fragment was then sequenced, and the exact physical co-ordinates were mapped using the BLAT tool of the UCSC Genome Browser.

## Results

### Clinical findings

**Fig. 2:**
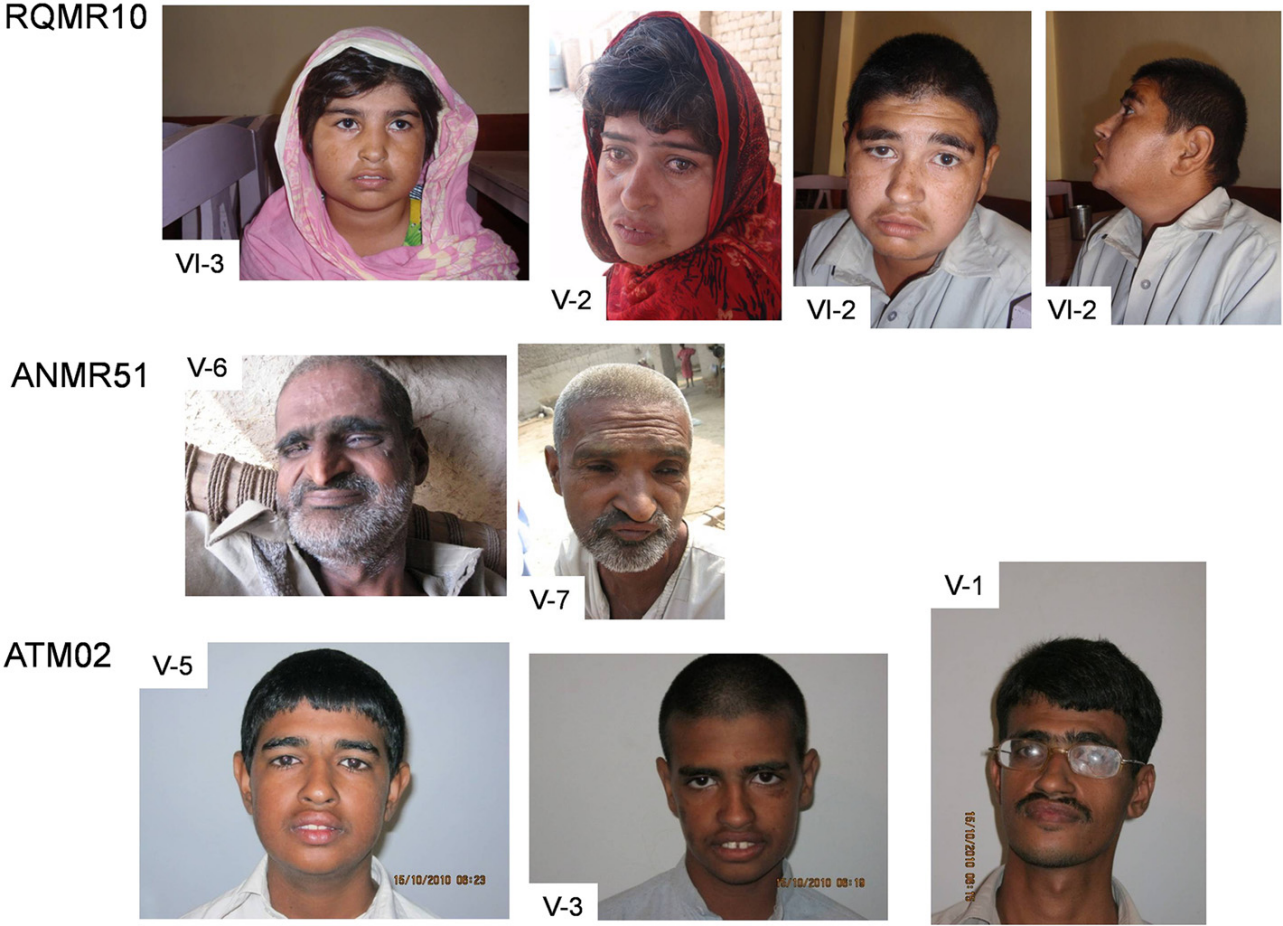
Photos of affected individuals from the three families

Families RQMR10 and ANMR51 (Figure 1): All affected, as well as several unaffected family members were examined clinically, with special attention to presence or absence of microcephaly, short stature, speech development, facial dysmorphisms, myopia, pigmentary retinopathy, pes planus, pes cavus, neutropenia and neuropsychiatric presentation, except for RQMR10 VI-1, who died of liver failure after joining the study, results shown in Table 1. Analysis of photographs (Fig. 2) by a dysmorphologist (J.S.) indicated for RQMR10 affected individuals having a boxy shaped face, straight eyebrows, mild synophrys, hypoplastic nares, prominent lips, and a small chin with mild prognathia (V-2, VI-3, VI-2), left eye exotropia (V-2, VI-3), ptosis (VI-2), slender nose with hypoplastic nares and a hanging columella (V-2). VI-2 also shows patchy skin hyperpigmentation over the temples, cheeks, nose, chin, and lateral aspects of the face and neck. For family ANMR51 individuals V-6 and V-7 have straight, bushy eyebrows with synophrys, deep-set eyes, short philtrum, and thin lips. V-6 also has a large chin, prominent nasal bridge with hypoplastic nares, pointed nasal tip and hanging columella. V-7 shows bilateral ptosis, exotropia, a broad nasal root and bridge and nasal tip with hypoplastic nares and hanging columella. Fundoscopy images for RQMR10 individual VI:2 were examined (E.H.), and showed abnormality compatible with a retinal dystrophy and with Cohen syndrome. The optic nerves were pale and the macular looked thinned and the blood vessels (arterioles) are attenuated (see Additional file 1: Figure S1). Night blindness and progressive vision loss were also observed in the affected individuals.

**Table 1:** Clinical features of affected members of families ANMR51, RQMR10 and ATM02. n.a. = not assessed

Family	ANMR51								RQMR10				ATM02		
Patient ID	IV-5	IV-4	IV-3	V-3	V-1	V-6	V-7	V-8	VI-2	VI-3	VI-1^a^	V-2	V-1	V-3	V-5
Sex	M	M	F	M	M	M	M	F	M	F	M	F	M	M	M
Age (Yrs)	25	27	17	20	15	>50	>50	>50	17	7	8	20	23	18	15
Neurodevelopment															
Developmental delay (motor, cognitive, speech)	Yes	Yes	Yes	Yes	Yes	Yes	Yes	Yes	Yes	Yes	Yes	Yes	Yes	Yes	Yes
Intellectual Disability	Severe	Severe	Severe	Severe	Severe	Severe	Severe	Severe	Severe	Severe	Severe	Severe	Moderate	Moderate	Moderate
Language	Single words	Single words	Single words	Single words	Single words	Single words	Single words	Single words	Single words	No speech	Single words	Single words	Small sentences	Small sentences	Small sentences
Autistic behaviors	N^b^	N^b^	N^b^	N^b^	N^b^	N^b^	N^b^	N^b^	N^bc^	N^b^	N^b^	Y	Y	Y	Y
Age 1^st^ walked	<2	<2	<2	<2	<2	<2	<2	<2	2	4	2	<2	2.5	2.4	2.0
Hypotonia?	n.a.	n.a.	n.a.	n.a.	n.a.	n.a.	n.a.	n.a.	n.a.	n.a.	n.a.	n.a.	yes	yes	yes
Coordination	poor	poor	poor	poor	poor	poor	poor	poor	poor	poor	poor	poor	poor	poor	poor
Facies															
OFC (cm)	49	49	47	n.a.	n.a.	47.5	48	48	n.a.	n.a.	n.a.	n.a.	51	49	49
Thick eyebrows	n.a.	n.a.	n.a.	n.a.	n.a.	No	Yes	n.a.	Yes	No	n.a.	No	Yes	Yes	Yes
Prominent nasal bridge	n.a.	n.a.	n.a.	n.a.	n.a.	No	Yes	n.a.	No	No	n.a.	No	Yes	Yes	Yes
short philtrum	n.a.	n.a.	n.a.	n.a.	n.a.	Yes	Yes	n.a.	No	No	n.a.	No	Yes	Yes	Yes
wide or wave shaped palpebral fissures	n.a.	n.a.	n.a.	n.a.	n.a.	Yes	No	n.a.	No	No	n.a.	No	Yes	Yes	Yes
Prominent incisors	n.a.	n.a.	n.a.	n.a.	n.a.	n.d.	n.d.	n.a.	Yes	Yes	n.a.	Yes	Yes	Yes	Yes
Mild synophrys	n.a.	n.a.	n.a.	n.a.	n.a.	Yes	Yes	n.a.	Yes	Yes	n.a.	Yes	Yes	Yes	Yes
Eyes															
Myopia	+	+	+	+	+	Blind	Blind	+	+	+	+	+	+	+	+
Retinopathy	n.a.	n.a.	n.a.	n.a.	n.a.	n.a.	n.a.	n.a.	+ retinal dystrophy	n.a.	n.a.	n.a.	n.a.	n.a.	n.a.
Neutropenia	n.a.	n.a.	n.a.	n.a.	n.a.	n.a.	n.a.	n.a.	-	Pan-cytopenia	n.a.	n.a.	+	n.a.	n.a.
Miscellaneous			Total loss of teeth		-	Prominent chin	-	-	Ptosis; hyperactivity			Pes planus & pes cavus; Truncal obesity	Pes planus hypoactive	Pes planus hyperactive recurrent skin infections	Pes planus; pica

^a^Now deceased

^b^these individuals were aloof, isolated, not sociable; however there were no repetitive behaviors

^c^Hyperactivity also noted

Some autistic-like traits were observed in affected members of RQMR10, who were reported to be aloof, isolated and not sociable; however in the context of their ID it was not possible to assess for autism spectrum disorder. No repetitive behaviors were noted. Based on clinical interview, the level of ID was estimated as moderate to severe in the affected individuals in RQMR10. In regards to social functioning, one female (V-2) was initially considered aloof, and later developed severe depressive disorder. RQMR10 VI-2 is reported as being hyperactive, having no sense of danger, and would frequently wander away from home.

Family ATM02 (Figure 1) is part of a much larger pedigree segregating with ID across many branches, with 18 affected individuals (see Additional file 2: Figure S2). In this study we were able to access just the portion of the family shown in Fig. 1. Three affected brothers presented with Cohen syndrome features (Table 1). In addition, individual V-1 had normal blood and urine tests, except for blood neutrophil at 2.0 × 10^−9^/L, and his brain MRI indicates non-specific mild to moderate brain atrophy with enlarged ventricles and subarachnoid spaces (see Additional file 3: Figure S3). However, due to lack of information on neutropenia and retinopathy, the diagnosis of COH1 was not made prior to the genetic investigation. They were all initially diagnosed with pervasive developmental disorder not otherwise specified (PDD-NOS). Ophthalmological examination later confirmed that the three affected brothers all present with pigmentary retinopathy.

Head circumference measurements (occipito-frontal circumference) were available for ANMR51 and ATM02, and show measures consistently > 3SD below the mean, suggesting that microcephaly is also a common feature in these families (Table 1).

### Mutation Detection in RQMR10, ANMR51 and ATM02 families

#### RQMR10 Family

**Fig. 3:**
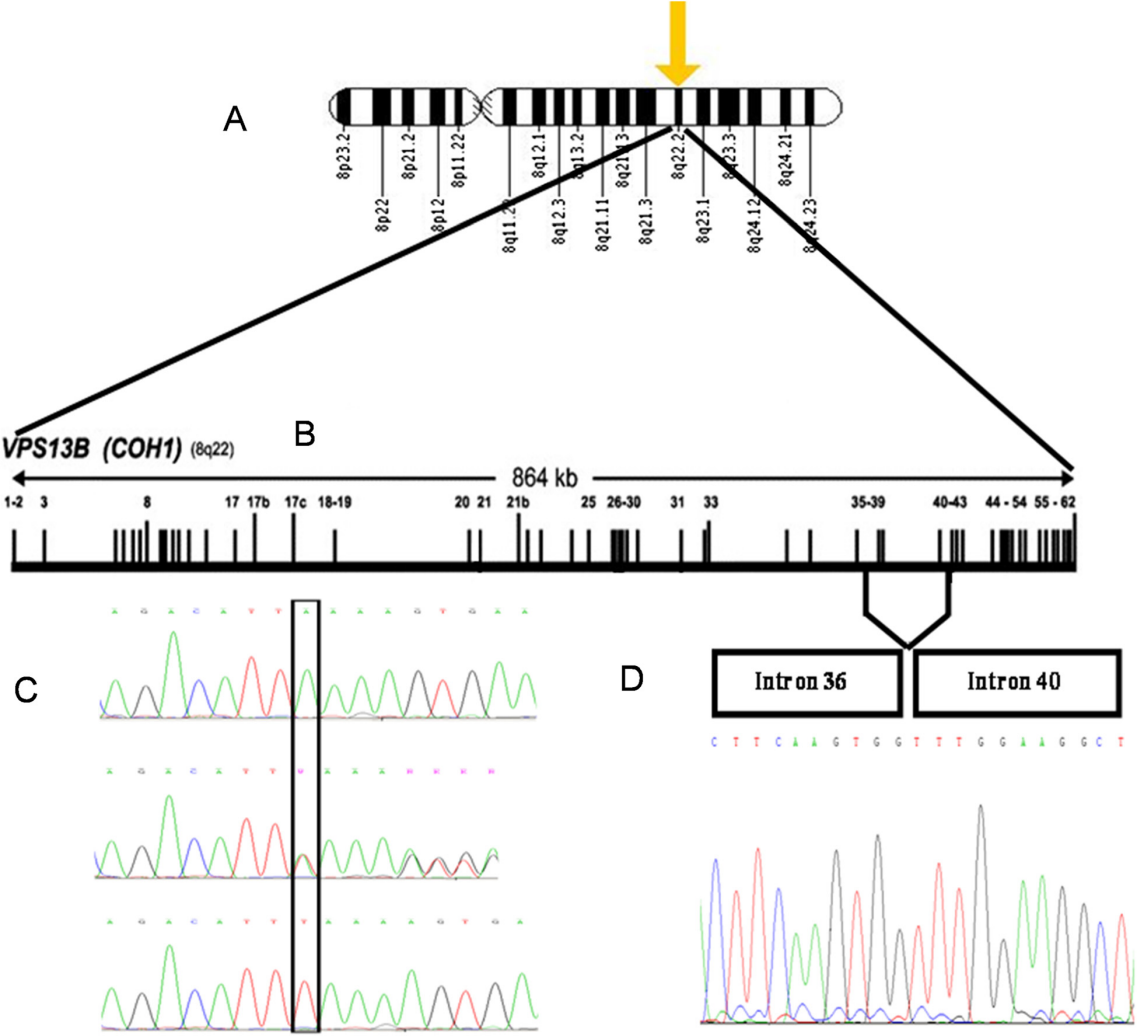
**a** Ideogram of chromosome 8, indicating location of HBD at 8q22. **b** Genomic organization of VPS13B/COH1, indicating location of the NM_017890.4:c.6879delT mutation (RQMR10 and ATM02) in exon 38, as well as the deleted region (exons 37–39) in ANMR51. **c** Electropherograms indicating the wild type homozygous (top), heterozygous (middle) and mutant homozygous (bottom) sequence across NM_017890.4:c.6879 (RQMR10 and ATM02). **d** Electropherogram from mRNA sequencing from an affected individual from ANMR51, indicating the (aberrant) splicing of exons 36 and 40

We identified only one ~25.430 Mb HBD region shared by the affected individuals, but not unaffected family members, on 8q22, flanked by SNPs rs1994035 and rs3098237 in family RQMR10. Sanger sequence analysis of all PCR amplicons revealed a deletion of 1 bp, T, (Fig. 3) at position chr8:100,732,719 (hg19) on chromosome 8q22.2. This deletion, NM_017890.4:c.6879delT, introduces a framseshift, resulting in premature protein truncation (p.Phe2293Leufs*24). Exon38 of *VPS13B* was sequenced in family members and in 293 normal unrelated healthy individuals from Pakistan. The homozygous c.6879delT mutation cosegregates with the clinical phenotype correctly within the family, and was not present in 293 healthy Pakistani controls. The variant is present as heterozygote in the Exome Aggregation Consortium Browser (ExAC; Cambridge, MA; http://exac.broadinstitute.org; Oct 2014), which includes over 8,000 exomes from South Asian individuals, but only one out of 122494 alleles total, and one out of 16,592 South Asian alleles.

#### ANMR51 Family

Analysis of genotypes for family ANMR51 revealed only one ~4.413 Mb HBD region shared by the affected individuals, but not unaffected family members, on 8q22 flanked by SNPs rs11783149 and rs2679749. Four consecutive exons (37–40) failed to amplify by PCR, yet amplified in unaffected family members, suggesting a possible deletion. The deletion fragment was confirmed and mapped by PCR amplification and sequencing of the junction fragment (forward primer-*VPS13B-JF-F-*5′-TCCTAAAATGTGCCA TTGGTT-3′; reverse primer-*VPS13B-JF-R*: 5′-TGCTGAAGAATAGATTGCTGAA-3′) (Fig. 3). Sequence analysis of the junction fragment showed the deletion happens between chr8:100,728,884 and 100,779,911 (hg19), spanning a region of 51,027 bp (hg19:g.chr8:100728884_chr8:100779911 del). Assuming utilization of the same acceptor and donor splice sites as for the wild type for the remaining part of the gene, the deletion is predicted to lead to a truncated protein, i.e., p.Gly2177Alafs*16. The deletion was co-segregating as homozygous mutation within the family. The deletion was not present in 293 unrelated healthy Pakistani individuals.

#### ATM02 family

Homozygosity mapping identified four HBD regions shared in three affected individuals: 1p36.32-p36.31 (rs10799181 to rs9308476, 1.87 Mb); 1p13.2-q21.3 (rs11804649 to rs6655975, 37.19 Mb), 8q21.11-q24.21 (rs7824974 to rs4407842, 52.49 Mb), and 16p13.3-p12.1 (rs2541696 to rs8047869, 24.27 Mb). Exome sequencing and analysis of two affected individuals (V1 and V5) led to the identification of the same 1 bp homozygous deletion at c.6879 T found in RQMR10. In the meantime, we have excluded any additional loss of function variant(s) (both homozygote and heterozygote) in genes known to carry pathogenic mutations for ID and ASD (SFARI gene list), in the two affected individuals, separately and jointly. The deletion at c.6879 T was confirmed by Sanger sequencing as homozygous in three affected individuals, but absent in unaffected family members and 293 controls, as mentioned for RQMR10.

### Haplotype sharing between RQMR10 and ATM02

**Fig. 4:**
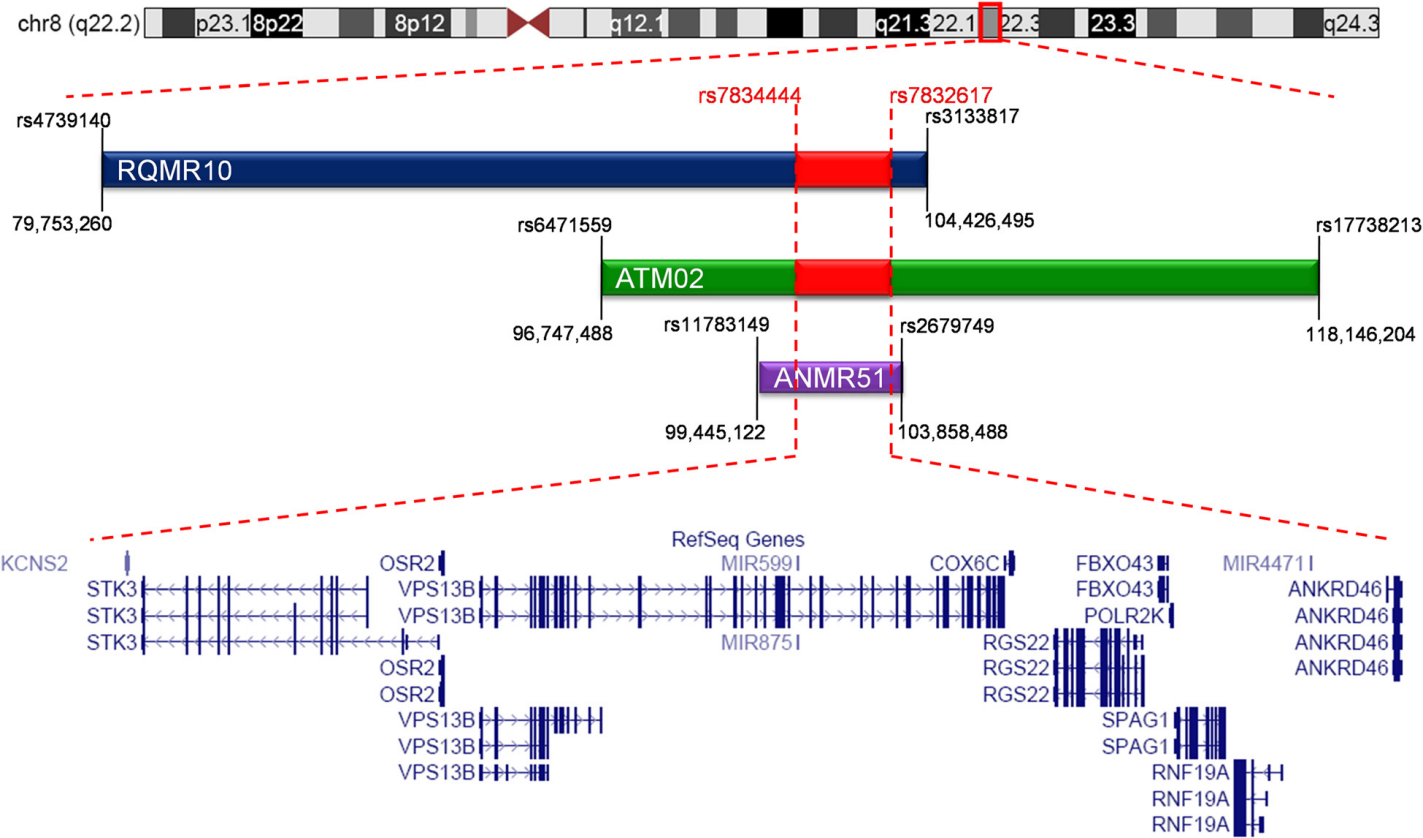
Homozygosity by descent of the Pakistani families and shared haplotype region for RQMR10 and ATM02. The homozygous-by-descent regions segregating with the phenotype within families RQMR10, ATM02 and ANMR51 are indicated with a dark blue, a green and a purple bar, respectively. The red bar indicates the region of shared haplotype for families RQMR10 and ATM02 and spans the entire *VPS13B*

Interestingly, RQMR10 and ATM02 were found to carry the same c.6879delT mutation independently. Subsequent joint analysis of shared HBD regions showed that among 592 common SNPs shared between Affymetrix and Illumina SNP chips within the overlapping HBD region on chr 8q22.2 in both families, 208 SNPs from a 2.14 Mb region showed the same haplotype (see in Fig. 4, also Additional file 4: Table S1), indicating a founder effect of this mutation within the population of Pakistan. Neither family is aware of any common ancestry, and has lived in regions separated by a distance of 534 km, although both are from the Baloch ethnic group. Based on the size of shared haplotype, the two families may share common ancestors approximately 50 generations ago (mean distance between the nearest flanking crossovers of the disease locus is 2/N morgans, where N is the number of meioses) [26].

## Discussion

We present a study of three large consanguineous Pakistani families with autosomal recessive ID and in some cases autistic behaviors, segregating with deletion mutations within the *VPS13B* gene. This large gene, spanning a region of 864 Kb with 62 exons, maps to chromosome 8q22.2 [7, 8]. The gene encodes a protein of 4,022 amino acids (plus a number of shorter isoforms) with several short regions showing homology with yeast vacuolar protein sorting-associated protein 13 (Vps13b), suggesting a possible role in vesicle-mediated protein sorting (VPS) and intracellular protein transportation [13, 14, 27, 28]. Functional studies show evidence for VPS13B co-localizes strongly with the *cis*-Golgi matrix protein GM130 [29]. The study also showed that VPS13B/COH1 is required for the maintenance of Golgi stacks as well as for reassembly of Golgi cisternae from ministacks, and can regulate the formation of membrane tubules from the Golgi. These functions suggest similarity with Golgins (Golgi autoantigens), except that VPS13B/COH1 does not contain coiled-coil domains. Absence or depletion of VPS13B/COH1 could lead to disruption in the Golgi complex integrity and functionality [29]. More recent work further demonstrated that VPS13B mutations, responsible for COH1, are associated with strong protein glycosylation defects, highlighting an important role for VPS13B in Golgi glycosylation and morphology, as well as in lysosomal–endosomal pathway maintenance [30].

The first mutation we report, a 51 Kb homozygous deletion encompassing four coding exons, assuming correct splicing of the flanking exons 36 to 41 during RNA maturation and processing, 793 bp of coding sequence would be deleted, leading to a shift in reading frame, with the substitution glycine to alanine at amino acid 2177, followed by a premature stop codon after a further 14 residues, leading to a 2191 amino acid residue protein (compared to 4022 for wild type, NM_017890). This premature stop codon would either lead to nonsense-mediated mRNA decay (NMD) or production of a truncated protein lacking a number of functional domains. This potential shortened protein (p.Gly2177Alafs*16) would lack domains DUF1162 and ATG_C (using SMART). The genomic region of the deletion contains no known segmental duplications, large repeats or other sequences that may be likely to predispose towards genomic instability (UCSC Genome Browser, hg19), and no CNVs have been identified at this region, according to the Database of Genomic Variants.

The 1 bp deletion at c.6879 T [NM_017890.4 (VPS13B_v001):c.6879delT], leads to a shift in frame and a premature stop codon (PTC) after amino acid 2315 (p.Phe2293Leufs*24). This premature stop codon would also either lead to NMD or production of nonfunctional protein. The truncated protein would lack many of the main predicted functional domains of the VPS13B protein, including Pfam predicted domains DUF1162 and ATG_C.

Since the two mutations would have a very similar functional effect, with truncation at similar points along the protein (2191 residues for RQMR10 and ATM02 mutation; 2315 residues for ANMR51 mutation), one might expect the resulting phenotype to be very similar. However, the affected individuals from these three families showed remarkable phenotypic variations, which could be due to other factors, including the degree of NMD that may occur for the two mutations, which in itself may depend on the nucleotide context of the premature stop codon and the assembly of the NMD complex [31]. Interestingly, most affected members of the two families with the c.6879delT mutation were either evaluated as having autism spectrum disorder (ASD) or of possessing autistic-like traits. Mutations in *VPS13B* have recently been found in additional cases of ASD or ID individuals with autistic features. Yu *et al.* in a study of 163 consanguineous and/or multiplex families with ASD through exome sequencing, found a homozygous frameshift mutation (Ser3945Glnfs*22) in a proband with ASD and mild dysmorphic features, as well as a rare missense mutation (p.S824A) in a proband with autism and mild facial dysmorphism and joint laxity [32]. In the same study, screening *VPS13B* gene in 612 families from the Simons Simplex Collection, one affected male child with ASD was found to be compound heterozygous for two different mutations in VPS13B (p.W963*/p.G2704R), and a second, unrelated male child affected with autism was compound heterozygous for two rare point mutations in VPS13B, p.S3303R and p.A3691T, both altering highly conserved residues of VPS13B [32]. Re-analyzing exome sequencing data of 488 ASD cases and 372 controls from the ARRA Autism Sequencing Collaboration (AASC) project, Ionita-Laza I *et al.* found two loss-of-function variants (one nonsense: p.Ser3383* and one splice site: c:2650 + 2 T > G), and a rare, homozygous probably-damaging variant (p.Arg3198Trp) in VPS13B that could contribute to autism risk [33]. Although only a single previous report of *VPS13B* mutations and COH1 in individuals of Pakistani origin has been published [34], identification of three unrelated large families from Pakistan carrying homozygous mutations in *VPS13B* indicates that this is an important gene, not only for COH1, but also for undiagnosed or misdiagnosed ID and ASD patients, in the Pakistani population. We believe that Cohen syndrome individuals with this “Baloch” variant may be enriched for ASD or autistic-like behaviors, which contrasts with the “friendly disposition” frequently associated with COH1. Early genetic screen for the extended families or clans, and local communities would be extremely useful in genetic counseling, and clinical diagnosis.

## Conclusion

We suspect that the c.6879delT mutation may be a common cause of COH1 and similar phenotypes among the Baloch population. Additionally, most of the individuals with the c.6879delT mutation in these two families also present with autistic like traits, and suggests that this variant may lead to a distinct autistic-like COH1 subgroup.

### Bioinformatic resources

1. Primer3 software (version 0.4.0): http://frodo.wi.mit.edu/primer3/
2. UCSC Genome Browser: http://genome.ucsc.edu/
3. Exome Aggregation Consortium browser (ExAC; Cambridge, MA): http://exac.broadinstitute.org
4. FASTQC: http://www.bioinformatics.babraham.ac.uk/projects/fastqc/
5. TMPRED: http://www.ch.embnet.org/software/TMPRED_form.html
6. SMART: http://smart.embl.de
7. SFARI gene: https://gene.sfari.org/

## Additional files

Additional file 1: Figure S1. Fundoscopy images for RQMR10 IV-2. Additional file 2: Figure S2. Extended pedigree of the Pakistan family ATM02. The region indicated in yellow highlight is included in Fig. 1 in the main article. Additional file 3: Figure S3. Brain MRI of ATM02 V-5. Additional file 4: Table S1. Shared haplotype between families RQMR10 and ATM02.
